# Re-do Operation Using a Robotic System due to Locoregional Recurrence after Initial Thyroidectomy for Thyroid Cancer

**DOI:** 10.1038/s41598-022-15908-x

**Published:** 2022-07-07

**Authors:** Dong Gyu Kim, Kwangsoon Kim, Ji-Eun Lee, Joon Ho, Jin Kyong Kim, Sang-Wook Kang, Jandee Lee, Jong Ju Jeong, Kee-Hyun Nam, Woong Youn Chung

**Affiliations:** 1 Department of Surgery, CHA Bundang Medical Center, CHA University, Seongnam, Republic of Korea; 2 Department of Surgery, College of Medicine, The Catholic University of Korea, Seoul, Republic of Korea; 3 Department of Surgery, Kosin University Gospel Hospital, Kosin University Colleage of Medicine, Busan, Republic of Korea; 4 Department of Surgery, Yonsei University College of Medicine, Seoul, Republic of Korea

**Keywords:** Surgical oncology, Thyroid cancer

## Abstract

Locoregional recurrent thyroid cancer is commonly treated with re-do operation. This study aimed to investigate the feasibility of using robotic system for re-do operation in locoregional recurrent thyroid cancer. Sixty-five patients who underwent re-do robotic operation using trans-axillary approach for locoregional recurrent thyroid cancer from October 2007 to April 2021 at Yonsei University Hospital were analyzed. Completion total thyroidectomy (CTT) was performed in 26 cases, CTT and modified radical neck node dissection (mRND) in 16, and mRND in 23. Most of the re-do robotic operations were performed at site of previous incision. All patients were diagnosed with papillary thyroid carcinoma (PTC). CTT with central compartment neck dissection (CCND) took 117.6 ± 26.3 min, CTT with mRND 255.6 ± 38.6 min, and mRND, 211.7 ± 52.9 min. Transient hypocalcemia occurred in 17 (26.2%) patients and permanent hypocalcemia occurred in 3 (4.6%). There was one case of recurrent laryngeal nerve(RLN) injury. One patient was diagnosed with structural recurrence after re-do robotic operation. Median follow-up duration was 50.7 ± 37.1 months. Re-do robotic operation can be an alternative for patients who are diagnosed with locoregional recurrent thyroid cancer after thyroidectomy, with no increase in morbidity, similar oncologic outcomes, and superior cosmetic satisfaction.

## Introduction

The global incidence of thyroid cancer has continued to steadily rise over the past two decades. Thyroid cancer is now considered as one of the most prevalent cancers in Korea. This is due to increased health check-ups and developed diagnostic modalities^1–3^. PTC is the most frequent subtype^4^. The standard treatment of thyroid cancer generally involves operation and postoperative treatments, such as radioactive iodine therapy (RAI) and thyroid-stimulating hormone (TSH) suppression therapy^3,4^. Thyroid cancers are more prevalent in women, whose concerns include cosmesis in the aspect of treatment^1–3^. The cosmetic consideration of reducing postoperative scars provided motivation for the development of various minimally invasive operations, even in patients with thyroid cancer. Endoscopic thyroidectomy (ET) started in the late twentieth century, while robotic thyroidectomy (RT) has been performed since 2007 and remains a commonly used method of remote access neck surgery at present^5,6^.

Chung et al. invented the new ET technique using a gasless trans-axillary approach in November 2001 at the Yonsei University hospital (Seoul, Korea)^7^. Trans-axillary approach robotic thyroidectomy (TART) was first performed in Yonsei University hospital thyroid cancer center in October 2007^6^. The qualified TART and even trans-axillary approach robotic mRND have been successfully performed in over 8000 cases^8,9^. Currently, not only eastern Asian groups, but western groups as well have reported the feasibility and oncologic safety of TART^10,11^.

Locoregional recurrence of PTC requires the recurred area node dissection and CTT^12^. Some of the concerns of re-do operation due to locoregional recurrence include cosmetic problems and operative difficulties due to adhesions. Therefore, performing robotic operation in locoregional recurrence can provide both local control of the tumor and better quality of life, including cosmetic effects. To the best of our knowledge, there is no study that has reported the operative outcomes of re-do robotic operation for locoregional recurrence. In this study, we analyzed and reported the feasibility and safety of re-do robotic operation for locoregional recurrence after initial thyroidectomy for thyroid cancer.

## Materials and methods

### Patients

There were 8243 patients who underwent TART using the da Vinci S, Si, Xi, or SP robotic system (Intuitive Surgical, Sunnyvale, CA, USA) from October 2007 to April 2021 at the Yonsei University hospital (Seoul, Korea). Of these patients, 7805 patients were diagnosed with a differentiated thyroid carcinoma and 425 patients were diagnosed with a benign tumor or benign thyroid disease. Re-do robotic operation was performed on 69 cases of those 7805 cases, and four cases were excluded in the study as immediate CTT, which is defined as an operation performed in less than two months after the first operation. Two of the four immediate CTT cases were follicular thyroid carcinoma (FTC) with wide capsule invasion and vascular invasion on the final pathologic report. Another case was a FTC with minimal capsule invasion and no vascular or soft tissue invasion on the final pathologic report; however, the patient wanted to undergo immediate CTT due to anxiety. Another case of CTT was performed due to a thyroid nodule, approximately 1 cm in size, found incidentally on preoperative ultrasonography (US). In the initial surgery, partial resection of the counter-lateral lobe was performed; however, in the final report, the benign-looking mass was diagnosed as PTC with central lymph node (LN) metastasis, prompting CTT. Finally, a total of 65 patients were enrolled in this study.

Of the 65 patients, an initial less than total thyroidectomy (LT) was performed on 42 patients, initial total thyroidectomy (TT) on 19 patients, and initial TT with mRND on four patients. All patients underwent ipsilateral CCND. Re-do robotic operation was performed in 26 patients as robotic CTT with CCND, in 16 patients as robotic CTT with mRND (one of the cases involved bilateral mRND), and in 23 patients as only robotic mRND. This study was conducted in accordance with the Declaration of Helsinki (as revised in 2013). This study was approved by the Yonsei University institutional review board (IRB No. 4-2021-0147), which waived the requirement for informed consent due to the retrospective nature of this study.

### Preoperative evaluation protocol

After initial thyroidectomy, postoperative care and follow-up was conducted according to the American Thyroid Association management guidelines^12^. For the detection of locoregional recurrence, patients underwent neck US and serum thyroid function testing at 6- or 12-month intervals. Each patient underwent chest radiography or computed tomography (CT) to detect potential lung metastasis. In cases of suspected recurrence in the remnant thyroid, resection bed, or LN, diagnosis was confirmed by US-guided fine needle aspiration. Patients also underwent neck CT and staging US for accurate preoperative evaluation of thyroid cancer aggressiveness including the tumor size, location, presence of extra-thyroidal invasion, multiplicity, and the presence of cervical LN metastasis.

### Operative procedures

The TART procedures have been previously described^6,13^. After the administration of general anesthesia, the patients were placed on the operating table in the supine position, with a soft pillow below their shoulders to allow slight neck extension. The lesion-side arm was raised and secured to expose the axilla. A 5–6-cm vertical skin incision was made on the axilla. A subcutaneous skin flap was created over the pectoralis major muscle and clavicle toward the midline of the anterior neck under direct visualization until identification of the sternocleidomastoid muscle (SCM). Subsequently, flap dissection was performed from the space between the sternal and clavicular heads of the SCM to the omohyoid and strap muscles, which were identified and drawn. The dissection progressed under the strap muscles until the ipsilateral lobe of the thyroid gland was exposed. At this point, an external retractor was inserted to raise the skin flap and muscles to establish a working space. The docking procedure for the robotic thyroidectomy was similar to that described previously^6,13^. Thyroidectomy was then performed in the same manner as conventional open thyroidectomy (OT).

The trans-axillary robotic mRND procedures have also been previously described^14,15^. The patients were placed on the operating table in the supine position, with a soft pillow below their shoulders to allow slight neck extension. The lesion-side arm was abducted to expose the axilla and lateral neck, and the head is rotated to face the contralateral side. A 7–8-cm vertical skin incision was made on the axilla, along the anterior axillary fold and the lateral border of the pectoralis major muscle. A subcutaneous skin flap was dissected medially over the SCM toward the midline of the anterior neck. Laterally, the trapezius muscle was identified and dissected upward along its anterior border. The dissection proceeded upward along the posterior surface of the SCM to expose the submandibular gland and the posterior belly of the digastric muscle. A wide external retractor was then inserted through the axillary incision to elevate the SCM. The docking procedure for the robotic mRND was similar to that described previously^14,15^. The mRND procedure was then performed in the same manner as open mRND.

**Figure 1:**
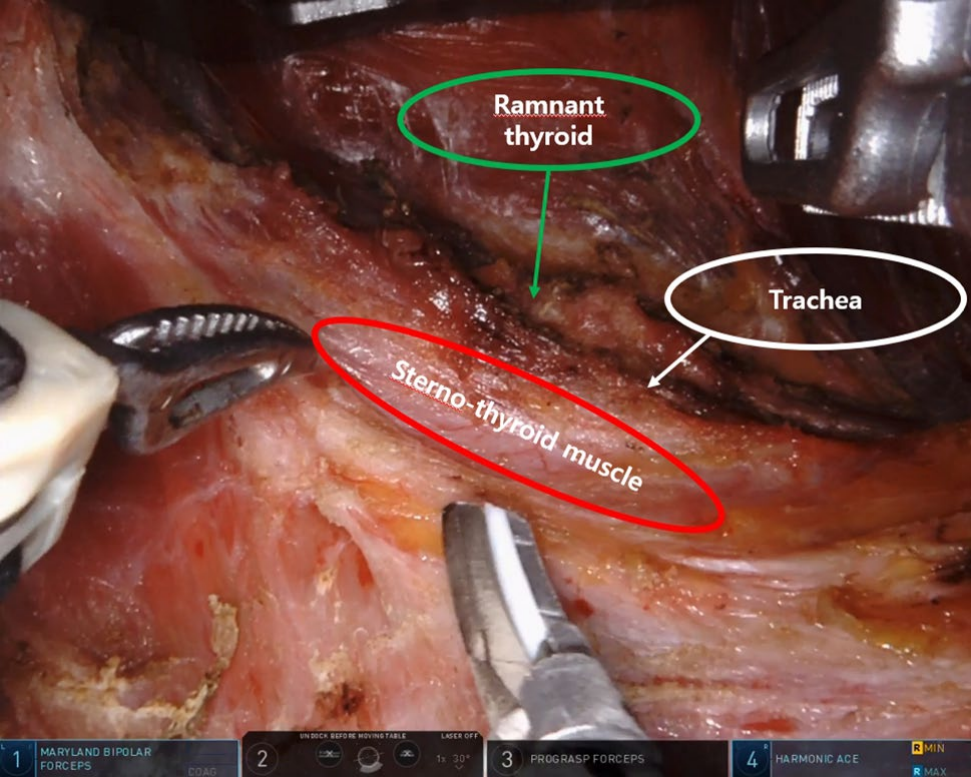
Completion total thyroidectomy robotic view. The patient was performed right side approach robotic right total thyroidectomy with central compartment neck node dissection previously. Re-do operation (completion total thyroidectomy) using robotic system for left thyroid recurrence was performed.

**Figure 2:**
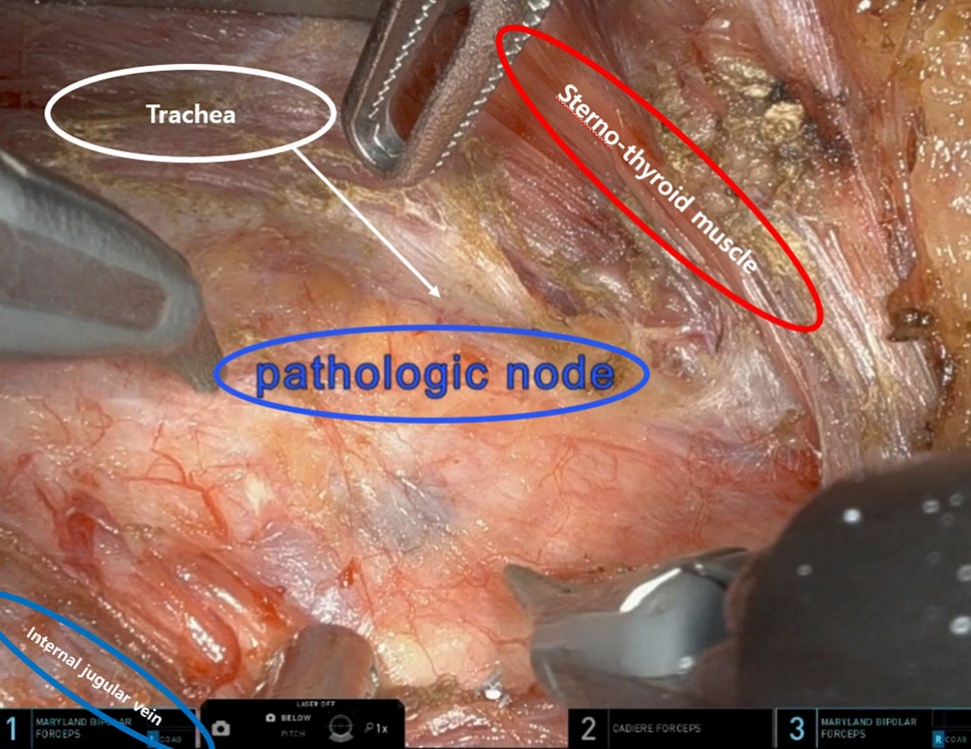
Para-esophageal lymph node dissection robotic view. The patient was performed left side approach robotic bilateral total thyroidectomy with central compartment neck node dissection and left modified radical neck node dissection previously. Re-do operation (para-esophageal lymph node dissection and right modified radical neck node dissection) using robotic system for right side para-esophageal lymph node and right lateral neck node recurrence was performed.

**Figure 3:**
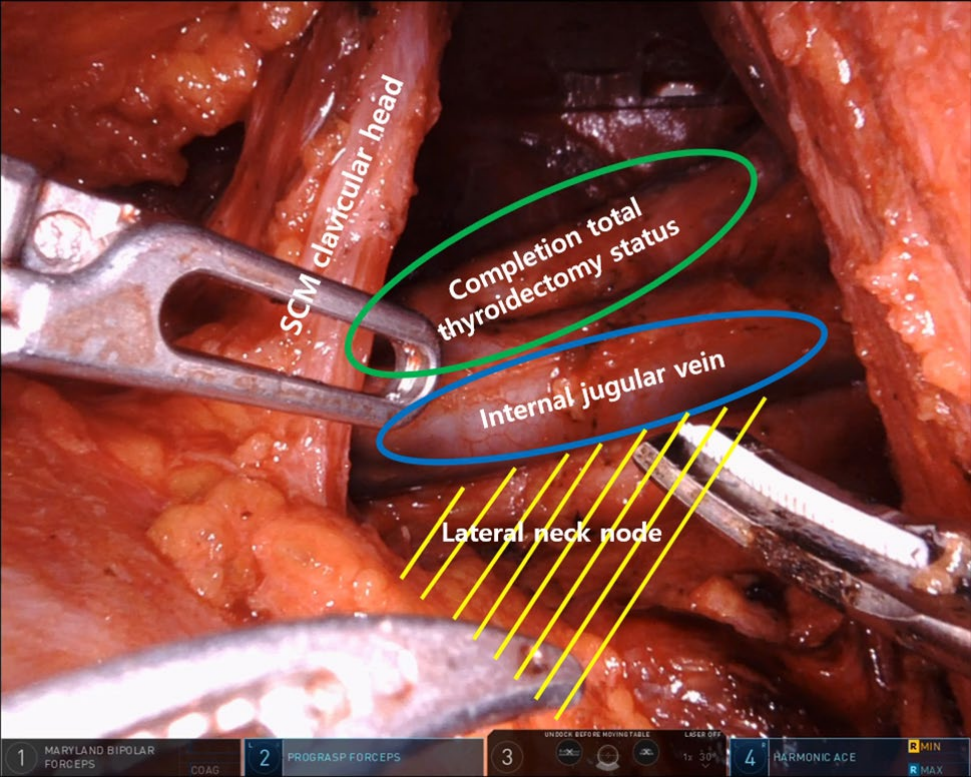
Modified radical neck node dissection robotic view. The patient was performed left side approach robotic left total thyroidectomy with central compartment neck node dissection previously. Re-do operation (completion total thyroidectomy and modified radical neck node dissection) using robotic system for left lateral neck node recurrence was performed. Abbreviations: *SCM* sternocleidomastoid muscle.

In the case of the previous incision approach completion total thyroidectomy, the skin incision was made along the exact previous incision. A subcutaneous skin flap dissection and SCM head dissection were done in the same manner as TART. A strap muscle dissection was performed between the sternothyroid and sternohyoid muscle. After strap muscle dissection, the trachea was identified and the remnant thyroid was dissected as counter-lateral lobectomy in robotic bilateral total thyroidectomy (see Fig. 1). If it is necessary to check the previous operation field, a strap muscle dissection was performed under the sternothyroid muscle in the same manner as in the original TART, with considerable care for RLN injury (see Fig. 2). In the case of the previous scar approach mRND, the skin incision was made along the previous scar, with an approximately 2 cm scar extension. The next procedure is similar to the trans-axillary robotic mRND method (see Fig. 3).

### Postoperative management and follow-up protocol

All patients were given suppressive doses of levothyroxine for TSH suppression immediately after operation. All patients underwent RAI ablation (30 mCi or 100 mCi or 150 mCi) 8–12 weeks postoperatively, and RAI whole-body scans were performed 5–7 days after RAI ablation. Serum thyroglobulin (Tg) concentration, anti-Tg antibody, and thyroid function test was evaluated, and neck US and CT scan were performed regularly during follow-up.

### Complication assessment

To evaluate postoperative complications, the parathyroid function was assessed by measuring serum calcium and intact parathyroid hormone (iPTH) concentrations at 1 week and 3 months postoperatively. Hypoparathyroidism was defined as a reduction in serum iPTH concentration below the normal limit with hypocalcemic symptoms such as paresthesia, spasm, tetany, tingling sensation, and even arrhythmia in severe cases^16^. Permanent hypocalcemia was diagnosed as persistent hypoparathyroidism status over 6 months after operation^17^. RLN injury was assessed through operation records and postoperative laryngoscope findings. Chyle leakage was defined as drainage fluid over 100 ml per day with high triglyceride level^18^.

### Statistical analysis

All statistical analyses were performed using the Statistical Package for the Social Sciences software for Windows version 23.0 (IBM Corp., Armonk, NY, USA). Continuous variables were reported as mean with standard deviation (SD), and categorical variables were reported as numbers with percentage.

### Ethics declarations

The study was conducted according to the guidelines of the Declaration of Helsinki and approved by the Institutional Review Board of Yonsei University (IRB No. 4-2021-0147; March 31th 2021).

### Informed consent statement

Patient consent was waived by the Institutional Review Board of Yonsei University due to the retrospective nature of the study.

## Results

### Baseline clinical characteristics of re-do robotic operation

Baseline clinical characteristics of the enrolled patients are shown in Table 1. The mean age of the patients was 39.3 ± 9.5 years. There were four male and 61 female patients. Initially, 18 patients were performed as OT, 13 patients as ET, and 34 patients as RT. Operation of 44 patients were performed at the Yonsei University hospital and that of 21 patients were performed at other hospitals. For the initial operation type, LT was performed on 42 patients, TT on 19 patients, and mRND with TT on four patients. Overall disease-free duration was approximately 62.8 ± 40.6 months; for LT patients, it was 60.8 ± 35.9 months; for TT with CCND patients, it was 63.4 ± 50.7 months; and for TT with mRND patients, it was 80.5 ± 39.1 months. For the re-do robotic operation, CTT with CCND was performed in 26 cases with an average operation time of 117.6 ± 26.3 min, CTT with mRND performed in 16 cases at 255.6 ± 38.6 min, and only mRND performed in 23 cases at 211.7 ± 52.9 min. CTT with CCND patients stayed in the hospital for 3.1 ± 0.7 days, CTT with mRND patients for 4.8 ± 0.5 days, and only mRND patients for 5.0 ± 1.5 days postoperatively. The same incision approach was allowed to be performed on 29 patients of initial LT with CCND and four patients of initial TT with CCND. Six patients of initial LT with CCND underwent re-do robotic operation with contralateral side approach owing to the surgeon’s preference. Two patients of initial TT with CCND and one patient of initial TT with mRND underwent re-do robotic operation with contralateral side approach owing to the contralateral side lateral LN metastasis. Bilateral approach re-do robotic operation was performed in one case wherein the patient initially underwent TT with CCND and experienced recurrence on both lateral neck node metastasis.

**Table 1:** Baseline clinical characteristics of re-do robotic operation.

Total 65 patients	
Age (years)	39.3 ± 9.5 (range 20–59)
Male:female	4:61 (1:15.3)
Initial operation access method (OT:ET:RT)	18:13:34 (27.7%:20.0%:52.3%)
Initial operation hospital (Yonsei University Hospital:other)	44:21 (67.7%:32.3%)
**Extent of initial surgery**	
LT + CCND	42 (64.6%)
TT + CCND	19 (29.2%)
TT + mRND	4 (6.2%)
**Disease free duration (months)**	62.8 ± 40.6 (range 6–220)
LT + CCND	60.8 ± 35.9 (range 6–156)
TT + CCND	63.4 ± 50.7 (range 7–220)
TT + mRND	80.5 ± 39.1 (range 43–120)
**Extent of 2nd surgery**	(Initial operation method OT/ET/RT)
CTT + CCND	26 (40.0%) ; (4/5/17)
CTT + mRND	16 (24.6%) ; (1/6/9)
Only mRND	23 (35.4%) ; (13/2/8)
**Operation time (min)**	
CTT + CCND	117.6 ± 26.3 (range 79–185)
CTT + mRND	255.6 ± 38.6 (range 206–340)
Only mRND	211.7 ± 52.9 (range 107–340)
**Approach direction**	
Same incision approach	33 (50.8%)
Contralateral side approach	9 (13.8%)
Others*	23 (35.4%)
**Postoperative hospital stay (days)**	
CTT + CCND	3.1 ± 0.7 (range 2–5)
CTT + mRND	4.8 ± 0.5 (range 3–5)
Only mRND	5.0 ± 1.5 (range 3–10)

Data are expressed as patient number (%) or mean ± SD.

*OT* open thyroidectomy, *ET* endoscopic thyroidectomy, *RT* robotic thyroidectomy, *LT* less than total thyroidectomy, *TT* total thyroidectomy, *CTT* completion total thyroidectomy, *CCND* central compartment neck node dissection, *mRND* modified radical neck dissection, *BABA* bilateral axillo-breast approach thyroidectomy.

*Others include one bilateral approach case due to bilateral neck node recurrence and not applicable cases such as initial open thyroidectomy or BABA approach thyroidectomy.

### Baseline pathological characteristics of re-do robotic operation

Baseline pathological characteristics of re-do robotic operation due to thyroid cancer are provided in Table 2. All patients were diagnosed as PTC. The mean tumor size was 0.8 ± 0.5 cm. Pathological T stage involved 35 cases of T0, 26 cases of T1, 2 cases of T2, 2 cases of T3, and no cases of T4. Pathological N stage had 18 cases of N0, 8 cases of N1a, and 39 cases of N1b. Two cases involved extra thyroidal extension and four cases were multi-focal cancer cases. Twenty patients among 23 were found to be BRAF-positive. An average of 4.1 ± 3.6 lymph nodes were retrieved in the central neck, and 34.2 ± 15.7 lymph nodes were retrieved in the lateral neck. An average of 1.3 ± 1.9 metastatic LN in the central neck and 4.4 ± 3.0 metastatic LN in the lateral neck were found.

**Table 2:** Baseline pathological characteristics of re-do robotic operation.

Total 65 patients	
**Pathology**	
PTC	65
Tumor size (cm)	0.8 ± 0.5 (range 0.3–2.3)
**pT stage**	
T0/T1/T2/T3/T4	35 (53.8%)/26 (40.0%)/2 (3.1%)/2 (3.1%)/0 (0%)
**pN stage**	
N0/N1a/N1b	18 (27.7%)/8 (12.3%)/39 (60.0%)
ETE	2/30 (6.7%)
Multifocality	4/30 (13.3%)
BRAF^V600E^ positive	20/23 (87.0%)
**Harvested LNs**	
CCND	4.1 ± 3.6 (range 1–20)
mRND	34.2 ± 15.7 (range 14–88)
**Positive LNs**	
CCND	1.3 ± 1.9 (range 0–8)
mRND	4.4 ± 3.0 (range 1–13)

Data are expressed as patient number (%) or mean ± SD.

*PTC* papillary thyroid carcinoma, *T* tumor, *N* node, *ETE* extra-thyroidal extension, *LN* lymph node, *CCND* central compartment neck node dissection, *mRND* modified radical neck dissection.

### Perioperative complication assessment

Perioperative complications of the re-do robotic operation were presented in Table 3. Transient hypocalcemia occurred in 17 patients and permanent hypocalcemia occurred in three patients. There were reports of one transient voice change case and one RLN injury case. There were four seroma cases and two chyle leakage cases, which did not need any invasive interventions. One patient needed percutaneous drainage for postoperative infection. The overall morbidity was 44.6% on re-do robotic operation.

**Table 3:** Postoperative complications of re-do robotic operation.

	Re-do robotic operation (n = 65)
Transient hypocalcemia (n/TT, %)	17 (26.2%)
Permanent hypocalcemia (n/TT, %)	3 (4.6%)
Transient voice change	1 (1.5%)
RLN injury	1 (1.5%)
Seroma	4 (6.2%)
Chyle leak	2 (3.1%)
Infection	1 (1.5%)
Overall morbidity	29 (44.6%)

Data are expressed as patient number (%).

*TT* total thyroidectomy, *RLN* recurrent laryngeal nerve.

### Surgical outcomes of re-do robotic operation

Surgical outcomes after re-do robotic operation were shown in Table 4. A total of 49 patients were treated with postoperative RAI, and all of them were revealed no abnormal uptake on the diagnostic whole-body scan. Under 1.0 ng/mL of serum Tg level without TSH elevation 3 months postoperative were seen in 57 patients (90.5%). There was one structural recurrence on a regular image study during regular follow-up at an out-patient clinic. Median follow-up duration was 50.7 months.

**Table 4:** Treatment outcomes of re-do robotic operation.

Total 65 patients	
Postoperative RAI ablation	49 (75.4%)
Ablation success based on DxWBS	49/49 (100%)
Suppression Tg < 1.0 ng/mL after 3 months	57 (90.5%)
Structural recurrence	1 (1.5%)

Data are expressed as patient number (%).

Average follow up duration 50.7 ± 37.1 months (range 3–121).

*RAI* radioactive iodine, *DxWBS* diagnostic whole body scan, *Tg* thyroglobulin.

## Discussion

A total of 1.1–28% of patients with PTC experienced recurrence after thyroid operation. Locoregional recurrence is the most common pattern of recurrence, which should be treated with re-do operation^19–22^. When performing re-do operation for locoregional recurrence, the previous scar is usually used. If the previous thyroidectomy was conventional OT, the same sub-platysma flap is also used. In most re-do open operations, adhesions from the previous surgery increases the difficulty of the operation^23^. Consequently, using the previous scar may cause cosmetic problems owing to the formation of a hypertrophic scar on the anterior neck as well as difficulty in swallowing due to postoperative anterior cervical adhesions^24^. Therefore, re-do open operation results in a patient’s low quality of life.

In recent years, the incidence of thyroid cancer in young and female patients has been increasing, and consequently, several patients now consider not only the treatment of thyroid cancer, but also their quality of life after operation, including cosmetic consideration^1–3^. Therefore, various operative techniques have been developed to improve quality of life. The TART allows a natural operative view similar to open operation and can help easily identify the RLN and parathyroid with a magnifying view, which is one of the advantages of robotic operation^6,8,9^. In addition, the greatest advantage is that CCND can be performed easily and accurately. There is also no interference of sight, without any camera disruptions caused by gas in the gasless method^13^. Furthermore, TART was proven to be better than OT when evaluating a patient’s quality of life postoperatively, such as reductions in neck sensory changes and swallowing discomfort after operation^25^. Re-do robotic operation should be considered in the case of recurrent thyroid cancer. Previously, we had not considered a re-do robotic operation due to difficulty. However, this has now become an option since it improves the patient’s quality of life. In this study, we analyzed the feasibility of re-do robotic operation for locoregional recurrent thyroid cancer.

Re-do robotic operation is able to satisfy the cosmetic demand of patients with recurrence^26^. In general, female patients tend to focus on cosmesis. They tend to choose the robotic thyroid operation 8.3 times more than male patients, despite female patients undergoing thyroid operation only 4.5 times more than male patients. In this study, female patients underwent re-do robotic operation 14.8 times more than male patients. Of the 18 patients who underwent OT initially, 14 needed mRND for lateral neck node metastasis, and they chose robotic mRND to avoid anterior neck scar extension. If patients have a scar on the axilla due to previous robotic or endoscopic operation, we were able to use that incision. If patients need to undergo mRND, only 2 cm extension was needed on the invisible area in the natural position, with surgical oncological safety^14^. During the re-do robotic operation, flapping of the lateral neck was not difficult due to a virgin surgical area. The central neck was slightly adhesive, but can still be dissected well and with care by experienced surgeons.

Regardless of the initial operation type, all re-do robotic operations were successful. Especially for cosmesis, 76.7% of trans-axillary wound patients did not need an additional scar. Among 65 patients, 43 patients had a previous axillary scar which were made from TART or trans-axillary approach ET. There were 22 patients without a previous axillary scar (ex. bilateral axillo-breast approach endoscopic or RT, OT) who needed an additional hidden axillary scar. One-third of 63 patients underwent initial operation at other hospitals and referred to Yonsei University hospital for a robotic operation. The average disease-free duration was 62 months, and 49.2% of the 65 patients experienced recurrence over 5 years after initial operation. Several surgeons may think that re-do robotic operations take longer than the initial operation due to postoperative adhesions. Kim et al. reported in their retrospective study of 5000 cases that the average operation time was 134.5 ± 122.0 min, and the average postoperative hospital stay was 3.3 ± 3.0 days^9^. Our study reported that the operation time lasted 117.6 ± 26.3 min, and the average postoperative hospital stay was 3.1 ± 0.7 days, which means that the re-do robotic operation did not take a longer time and did not need longer postoperative hospital stay.

Some patients may experience postoperative complications after thyroidectomy: hypoparathyroidism, RLN injury or bleeding, and infection. The two most common complications of thyroidectomy are hypocalcemia and RLN injury^27^. Christou et. al reported that hypocalcemia can occur in 20 ~ 30% and RLN injury can occur in 5 ~ 11% of the patients^27^. Lorente et. al reported that transient hypoparathyroidism occurs in 42.3% of the patients and permanent hypoparathyroidism in 4.6%^28^, while Pisanu et. al reported that transient RLN injury can occur in 3.5% of the patients and permanent RLN injury can occur in 1.2%^29^. In this study, transient hypocalcemia occurred in 26.2% and permanent hypocalcemia in 4.6%. Patients with permanent hypocalcemia did not need additional treatment aside from oral calcium carbonate supplement, as needed. It is consistent with other studies even though this data is from the re-do operation. There was one case of transient vocal cord palsy and one of RLN injury, with the results also similar to other published data^27–29^. There were two chyle leakage cases that needed conservative care; these patients were discharged without any additional procedure. One of the greatest advantages of TART, which is the magnifying view, could result in keeping the parathyroid gland and avoiding RLN injury during dissection^7,13,14^. The incidence of complications is higher in advanced cases^27^. However, while unexpected RLN or internal jugular vein invasion had been occasionally observed during the initial operation, no patient enrolled in the current study had RLN or IJV invasion due to the prompt surgical intervention after the diagnosis of recurrence.

There were 49 patients (75.4%) who needed postoperative radioactive iodine ablation, and all were successful. There were six patients with suppressive Tg over 1.0 ng/mL for 3 months after re-do robotic operation; these patients had no structural recurrence on image study during the follow-up period. There was one case of structural recurrence. A 34-year-old male patient who had undergone TART (left total thyroidectomy with CCND) 76 months before counter-lateral thyroid recurrence underwent robotic CTT with CCND. At 42 months after re-do robotic operation, imaging study showed suspicious nodules on the left at levels 3 and 4 and on the right at levels 4 and 6 areas. Fine needle aspiration biopsy on the left level 4 revealed it as a metastatic thyroid cancer. A mRND was planned, but patient was lost to follow-up due to his transfer to another hospital.

BRAF mutation studies were carried out via real-time PCR of tumor specimens, as well as BRAF^V600E^-specific staining of tumors. A positive result confirmed a BRAF mutation. Approximately 50%-70% of BRAF mutations are reported in Korean patients with thyroid cancer^30,31^, and the role of BRAF mutation in the aggressiveness of PTC and the prognosis of patients remains controversial. Some studies have shown that BRAF mutation is associated with worse clinicopathological risk factors, higher TNM staging, and thus, increased risk of recurrence^32–34^. In the present study, BRAF mutation studies were performed in 21 patients, and 18 (85.7%) patients were positive. Our results suggest that the presence of a BRAF mutation may be significantly associated with locoregional recurrence. However, other studies have reported that BRAF mutations might not be an important factor for prognosis in patients with PTC^31,35^. Other concurrent factors, such as TERT or RET/PTC mutations, must be further investigated.

The limitation of this study is that it is a retrospective study that depends only on medical records. There may also be selection bias owing to it being a single-center study. We also could not evaluate any objective indicator for a patient’s quality of life. One of the biggest advantages of robotic operation is the improvement in the patient’s postoperative condition, including reduction in postoperative pain, less sensory changes, better voice and swallowing functions, and higher cosmetic satisfaction^13,25^. A small sample size is also one of the weak points of this study. We tried to compare with the open operation due to locoregional recurrence, but the difference between the two groups is heterogeneous, and the open operation group size is 10 times more than that of the robotic operation group. After more data collection, the comparison between re-do robotic operation and re-do open operation can be analyzed, and quality of life assessment can also be evaluated in future studies.

Despite several limitations, this is the first study that evaluated the use of re-do robotic operation due to locoregional recurrence after initial thyroidectomy with thyroid cancer. However, there are already a few articles for robotic operation due to locoregional recurrence. Some of these articles include case reports on oropharyngeal cancer or prostate cancer and a meta-analysis of oropharyngeal cancer with trans-oral robotic operation^36–39^. One of the strengths of this study is that it was executed under a single center, which is considered to be the largest robotic thyroidectomy center in the world, with a uniform quality of data.

## Conclusions

To the best of our knowledge, this is the first study that has reported the feasibility and safety of re-do robotic operation. Re-do robotic operation can be an alternative operative option for patients who are diagnosed with locoregional recurrence after thyroidectomy with no increase in morbidity, similar oncologic outcomes, and superior cosmetic satisfaction.
